# Examining the effectiveness of financial vs. social incentives to participate in a smartphone ecological momentary assessment well-being study: protocol for a randomized controlled trial

**DOI:** 10.3389/fdgth.2026.1742191

**Published:** 2026-06-12

**Authors:** Abdur-Rahman Ridwan, Lesley-Anne Carter, Jack S. Benton, Jamie Anderson

**Affiliations:** 1 Cathie Marsh Institute for Social Research (CMI), Department of Social Statistics, School of Social Sciences, The University of Manchester, Manchester, United Kingdom; 2 Centre for Biostatistics, Division of Population Health, Health Services Research & Primary Care, School of Health Sciences, The University of Manchester, Manchester, United Kingdom; 3 Department of Geography, School of Environment, Education and Development, The University of Manchester, Manchester, United Kingdom

**Keywords:** ecological momentary assessment, experience sampling method, incentives, mobile app, momentary well-being, study feedback, prize draw, vouchers

## Abstract

Ecological momentary assessment (EMA) enables the collection of real-time data on individuals’ feelings, behaviors, and contexts through repeated short assessments, typically delivered via smartphones. Despite its value for understanding everyday determinants of well-being, EMA is less commonly implemented than traditional, retrospective self-report methods in many applied and community-based research settings, in part because of the participant burden it can impose. This study protocol describes a randomized controlled trial designed first to estimate invitation-stage willingness to participate in an explicitly described smartphone-based EMA study recruited through a public community platform, and second to evaluate whether scalable financial and social incentives increase this willingness in this setting. Specifically, the study investigates how a lottery-based financial incentive and a non-financial social incentive influence willingness to participate (Yes/No) in an explicitly described EMA study focused on the impact of urban environments on subjective well-being. It also examines reasons for non-participation among those unwilling to participate, as well as alternative incentive preferences that could motivate future participation. The study targets 4,809 users of a digital community platform for residents, workers, and visitors in King's Cross, London. Using a stratified block randomization procedure with randomly permuted block sizes, participants are allocated to one of three conditions: a financial incentive (prize draw for shopping vouchers), a social incentive (invitation to a study feedback event), or a control condition (no incentive). Randomization is stratified by residential status. Invitations describing the EMA study in the neighborhood are sent to user email addresses and willingness-to-participate responses are collected through a brief follow-up questionnaire. Logistic regression will test differences across groups, while secondary analyses will explore subgroup variations by age, gender, and residential categories, as well as reported reasons for non-participation and preferred incentive alternatives. This trial is expected to provide evidence on invitation-stage willingness to participate and on the effectiveness of scalable financial and social incentives for promoting participation in EMA-based well-being research. Together with insights into reasons for non-participation and preferred incentive alternatives, these findings will inform scalable approaches to public engagement with smartphone-based EMA in health and well-being studies.

## 1 Introduction

Subjective well-being (SWB) refers to how people experience and evaluate their lives and specific domains and activities in their lives. Interest in SWB has gained increasing attention across economic, social, and health research because it provides insight into the conditions under which people live and can inform policy decisions in these domains (1–3). SWB includes both evaluative and affective dimensions: evaluative SWB concerns people's overall assessments of their lives, typically through life satisfaction, whereas affective well-being concerns the feelings, emotions, or states they experience over time (3). While overall life satisfaction provides an important global assessment, SWB is also shaped by short-term dynamics and by the context in which people live, work, and move. In particular, affective SWB is inherently dynamic and context-dependent, which creates a need for research approaches that can measure lived experience as it unfolds in everyday life rather than relying solely on retrospective self-reports (4, 5).

Ecological Momentary Assessment (EMA), also referred to as the Experience Sampling Method (ESM), is one such approach to capture momentary changes in SWB (6, 7). EMA is a self-report data collection that uses signals (e.g., notifications) to prompt individuals to report their behaviors, moods, perceptions, and experiences in their natural environment (6–8). Participants are typically prompted on smartphones to complete brief questionnaires multiple times per day while engaging in their usual activities (8, 9). By capturing data in real time, EMA reduces recall bias common in traditional survey methods and enables the investigation of temporal associations and variability in experience as they naturally unfold (8, 9). Compared with global self-report measures of evaluative well-being, EMA is particularly well suited to assessing affective SWB because it captures moods and emotions in their immediate context (9). These features also make EMA especially useful for studying how neighbourhood environments relate to well-being. Growing evidence indicates that the built environment of local neighbourhoods influences residents’ well-being (10–12), and EMA offers a useful way of examining these relationships by capturing momentary affective SWB together with *in-situ* information on activities, perceptions, and environmental context (13, 14).

Despite these advantages, the successful implementation of EMA depends on individuals' willingness to participate in intensive, repeated self-reporting (15). Protocols that require frequent prompts, longer study duration, or longer questionnaires may be perceived as burdensome; evidence indicates that higher assessment intensity can increase perceived burden and reduce adherence or compliance (16–18). This matters not only for data completeness after enrolment, but also for who participates in the first place, as willingness to take part may vary systematically across individuals (15).

Incentives are commonly used in EMA studies to encourage participation. Evidence reviews of mobile EMA sampling describe several types of compensation structures, including fixed payments, response-contingent rewards, raffles or lotteries, and studies offering no reward (19). However, most empirical evaluations of incentives in EMA have focused on engagement after enrolment, such as prompt completion or compliance, rather than on initial uptake at the invitation stage (20, 21).

Evidence on how study design features influence initial participation in EMA remains limited. One experimental vignette study found that higher guaranteed compensation increased reported willingness to participate in a hypothetical EMA study (22). While such designs provide useful insights into how potential participants evaluate study features, they rely on stated intentions rather than actual behavior. More recent research has examined participation using staged recruitment processes. For example, a population-based study documented substantial attrition across a postal recruitment funnel and showed that uptake varied when invitees were presented with protocols differing in burden (23). However, this does not reflect real-world digital health contexts in which invitations are delivered through apps or online platforms, and participation decisions are made in response to brief digital messages rather than multi-stage postal recruitment (23).

It is also important that recruitment strategies are feasible to implement at scale and under practical resource constraints. Evidence from survey methodology suggests that monetary incentives generally increase response rates more than non-monetary incentives, that prepaid incentives often outperform promised incentives, and that money may be more effective than vouchers or lotteries, although prepaid incentives may be difficult to administer in web-based surveys or recruitment contexts (24, 25). At the same time, lottery-based incentives are commonly used within EMA studies to sustain participant engagement after enrolment, as they provide a salient reward while lowering expected marginal costs (19). Whether such incentives also influence individuals' initial decisions to participate, however, remains less clear.

Non-financial strategies may also encourage participation, particularly in community-based research where reciprocity and shared benefit—such as providing feedback on study findings—may motivate engagement. Behavioral theories suggest that different incentives may appeal to different motivations, including financial rewards, topic relevance, or perceived community benefit (26, 27). However, there is limited empirical evidence on whether such non-financial incentives are effective in improving participation in EMA studies.

To address this evidence gap, this study will evaluate the effectiveness of two scalable incentive strategies—a lottery-based financial incentive and a community-framed non-financial incentive—in encouraging invitation-stage willingness to participate in a future EMA study among an urban neighbourhood population. A guaranteed payment incentive arm was considered but not included because the primary methodological question is whether implementable, lower-cost alternatives can improve recruitment in a community platform context. To ensure the study still informs this design space, we will also assess nonparticipants' stated preferences for alternative incentive structures, including guaranteed payments (see Supplementary File S1).

The study has five objectives:

1. To estimate the overall invitation-stage uptake for the proposed EMA study (i.e., the proportion of invitees who register interest) in this community recruitment context.
2. To determine whether offering a financial incentive (eligibility for one of four £100 prize draws) or a social incentive (exclusive invitation to a neighbourhood social event for sharing study results) increases individuals’ willingness to participate in experience sampling research, compared with offering no incentive.
3. To investigate whether the effectiveness of the incentives in promoting participation varies across demographic and residential subgroups, including residential status, age group, and gender.
4. To examine the reasons provided for non-participation and assess whether the likelihood of endorsing specific reasons differs by incentive condition among those who declined participation.
5. To identify which alternative incentives are most preferred by individuals who declined participation, and assess whether these preferences differ by the incentive condition originally offered.

## 2 Methods

The study methods are reported in accordance with SPIRIT guidelines (participants, interventions, outcomes, randomization, data collection and management, analysis, and monitoring) (28).

### 2.1 Study design

This is a three-arm superiority randomised controlled trial (RCT) that evaluates the impact of two types of incentives against a control condition on willingness to participate in EMA research among an urban neighbourhood population.

### 2.2 Study setting

This study is conducted in the King's Cross, an urban neighbourhood in London, United Kingdom. The specific setting is the regenerated area within the King's Cross neighbourhood. The once derelict area has been transformed into a vibrant new part of London that currently has a mixed-use development with leisure and community facilities, shops, homes, offices, restaurants, bars, galleries, music venues, and an arts college. Table 1 presents the demographics of King's Cross neighbourhood.

**Table 1:** 2021 census topic summaries, ONS © crown copyright. Created by: Insight & Improvement, Strategy Family, © LB Camden.

Demographics		Number	%
Ethnic Groups	White	4,617	45.4%
	Asian or Asian British	3,233	31.8%
	Black or Black British	985	9.7%
	Mixed/Multiple Ethnic Groups	717	7.0%
	Other Ethnic Group	628	6.2%
All Usual Residents Sex	Male	4,861	47.7%
	Female	5,322	52.3%
All User Residents Resident Type	Lives in a household	7,853	77.1%
	Lives in a communal establishment	2,328	22.9%
All User Residents (16+) Education	Level 4 qualifications and above	3,975	43.4%
	Level 3 qualifications	2,501	27.3%
	No qualifications	1,135	12.4%
	Level 2 qualifications	616	6.7%
	Level 1 & entry level qualifications	464	5.1%
	Other qualifications	284	3.1%
	Apprenticeship	179	2.0%
Age groups	15-24 years	3,502	34.4%
	25-44 years	2,972	29.2%
	45-59 years	1,446	14.2%
	60-74 years	910	8.9%
	5-14 years	644	6.3%
	0-4 years	332	3.3%
	75-84 years	278	2.7%
	85 + years	99	1.0%
Employees^a^	Full-time employees	15,000	71.43%
	Part-time employees	6,000	28.57%

a Employees are not necessarily residents in King's Cross. Some of them reside outside King's Cross. Furthermore, people could have more than one job. Data is retrieved from ONS Business Register and Employment Survey. From http://www.nomisweb.co.uk.

### 2.3 Participants

The participants in this study are visitors, residents, and commercial occupiers in the regenerated area of King's Cross. Commercial occupiers could be classified as office occupiers or retail occupiers. Office occupiers have large office spaces in the neighborhood; examples are Google, Meta, Universal Music, PRS for Music, and Havas. Retail occupiers have smaller spaces, and they include independent stores, gyms, hair and beauty salons among others. Employees working in either office or retail occupiers are “commercial occupiers” and comprise the first category of participants in this study. The office-vs.-retail distinction is provided for descriptive context only; for the purposes of this study, all employees working for non-residential occupiers were grouped as “commercial occupiers.” Secondly, residents are household owner-occupiers, private renters, and social housing occupants in the neighbourhood. Lastly, visitors are individuals who occasionally or regularly travel or visit King's Cross.

#### Inclusion criteria

- Registered on the King's Cross community smartphone application.
- App users living, working, or have previously visited King's Cross
- Consented to receiving email communications during their smartphone app sign-up

#### Exclusion criteria

- Users of the King's Cross community smartphone app who have not given consent to receive email communication about the neighbourhood.

### 2.4 Sample size

The sample size for this study was fixed at 4,809 individuals, representing the total number of users in the King's Cross Equiem database who met the eligibility criteria. Power analysis was conducted to evaluate the study's ability to detect differences between the three incentive conditions under varying levels of willingness to participate in the control group. Because the total N was fixed by the sampling frame, results are presented as minimum detectable differences (MDEs) in percentage points for each assumed control rate (29). Power for differences in proportions was calculated using standard two-sample methods for binary outcomes (30).

Due to limited directly comparable external estimates of baseline participation without incentives in similar digital-based recruitment contexts, a plausible range of control proportions (1%–70%) was assumed and used as a sensitivity analysis. Because EMA recruitment can yield single-digit participation rates in some settings (23), MDEs for low baseline proportions (1%–10%) are included within this range as part of the same fixed-N sensitivity framework. Power calculations were then conducted for each assumed proportion, using a Bonferroni-adjusted significance level of 0.01667 (0.05/3) to maintain a family-wise error rate of 0.05 across the three planned pairwise comparisons among the three conditions (31). Table 2 summarizes the assumptions and the corresponding minimum detectable differences.

**Table 2:** Results of power analysis for detecting differences in willingness to participate across incentive conditions.

N	Proportion willing in control	Percentage point difference
4,809	0.01	1.16
4,809	0.02	1.53
4,809	0.03	1.81
4,809	0.04	2.04
4,809	0.05	2.24
4,809	0.06	2.41
4,809	0.07	2.57
4,809	0.08	2.72
4,809	0.09	2.85
4,809	0.10	2.97
4,809	0.20	3.86
4,809	0.30	4.36
4,809	0.40	4.61
4,809	0.50	4.66
4,809	0.60	4.52
4,809	0.70	4.18

With a fixed sample size of 4,809 and using the adjusted alpha for planned pairwise comparisons described above, the study would have approximately 80% power to detect absolute differences in willingness to participate of a few percentage points, depending on the assumed control proportion (Table 2). For example, if 30% of individuals in the control group were willing to participate, there would be sufficient power to detect an increase of 4.36 percentage points, corresponding to a willingness rate of 34.36% in an incentive condition. By contrast, if baseline willingness is in the single-digit range, detecting smaller absolute increases (e.g., 2 percentage points) with 80% power at *α* = 0.01667 would require a substantially larger sample per group than was available under the fixed sampling frame. These MDE benchmarks will be used to interpret observed effects under the predetermined sampling frame. If baseline willingness is in the single-digit range, smaller absolute effects may not be detected reliably; accordingly null findings will be interpreted relative to the corresponding low-rate MDE. These detectable differences provide a transparent benchmark for interpreting effects under the predetermined sampling frame.

### 2.5 Recruitment

The three groups of participants: commercial occupiers, residents, and visitors to Kings Cross are recruited from a neighbourhood mobile application with registered users. The participants could consequently be considered as a self-selected subsample of the neighborhood's population. The mobile application is the King's Cross Community App, and it is available on Android through Google Play and iPhone through the App Store (32). It is used to share information about the neighbourhood salons, bars, shops, and restaurants, in addition to relevant deals, offers, events, and ticket giveaways in the neighbourhood. The app is informational and does not support peer-to-peer communication (e.g., user posts or direct messaging), and users cannot view or identify other registered users. Table 3 presents the composition of the three groups of participants to be recruited in the mobile app.

**Table 3:** Total composition of king's cross community mobile application users.

Group	Total app users	Recruited no.	Recruited no. (%)	Total recruited (%)
Commercial Occupiers (Office & Retail)	3,869	1,172	30.29	24.37%
Residents	1,208	541	44.78	11.25
Public (Visitors)	11,176	3,096	27.7	64.38
Total	16,253	4,809		100

The King's Cross mobile app is owned by The King's Cross Group, a UK property developer specializing in mixed use development with a focus on placemaking and regeneration. This urban regeneration specialist has led the regeneration of King's Cross for more than two decades and remains the asset manager for the neighbourhood. The application is managed by Garden State London—a marketing and digital communications agency company—to disseminate latest information about the neighbourhood to registered users on the app and emails. Study invitations and intervention emails were distributed to registered users via email (rather than being posted within the app), and no study-related content was shared through the app interface. The agency is responsible for recruiting participants for this study and delivering the study intervention to eligible participants following the participant eligibility criteria and other guidelines provided by the study authors.

### 2.6 Trial flow

**Figure 1:**
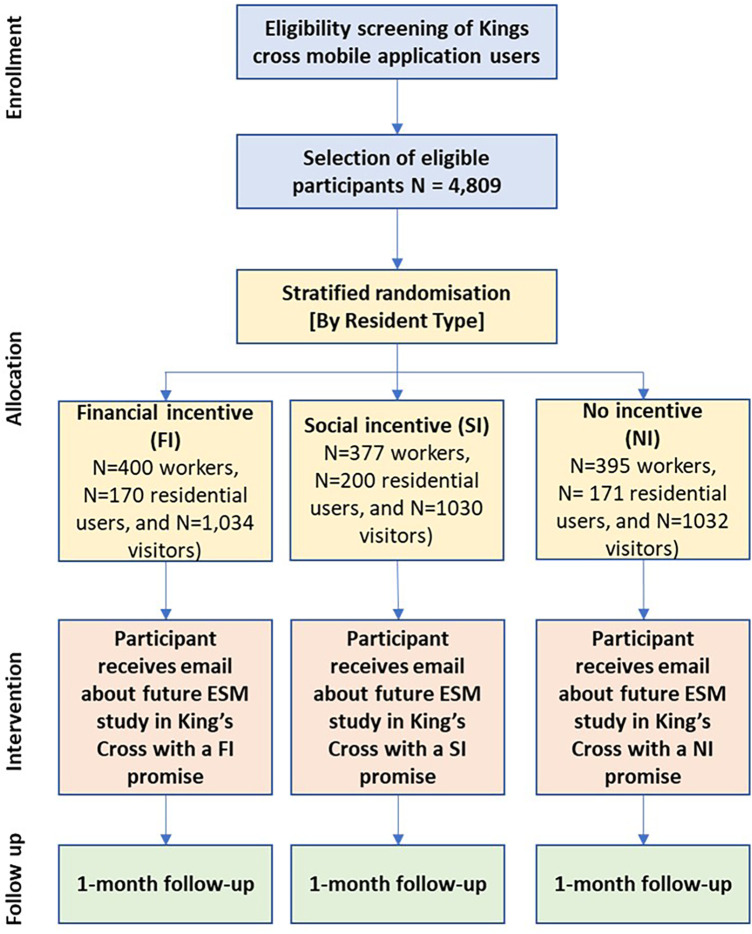
Schedule of enrolment, allocation, invitation delivery, reminder, and outcome ascertainment. **Note**. All invitees had previously consented to receive email communications during sign-up for the community app or the King's Cross website email newsletter. Additional informed consent for study participation was obtained at questionnaire entry before any survey items were answered. Reminder emails were sent only to nonresponders.

The trial commenced on September 12, 2024, and ended on November 30, 2024. Participants in different neighbourhood strata received randomized emails simultaneously (Figure 1). The initial email was disseminated on September 12, 2024, and the participation survey link remained open until November 30, 2024. A single follow-up (reminder) email was disseminated one month later (October 17, 2024).

### 2.7 Randomization

#### 2.7.1 Sequence generation

Participants were randomized into three incentive groups (financial incentive, non-financial incentive, or no incentive) using a stratified block randomization procedure. Stratification was based on participant type within the Equiem communication platform (residents, visitors, and commercial occupiers). Randomization lists were generated using the Sealed Envelope online randomization service, which employed randomly permuted block sizes of 3, 6, and 9 to ensure a balanced allocation across incentive groups within each stratum. Sealed Envelope is a web-based clinical trial randomization platform that generates allocation sequences based on user-specified parameters (e.g., allocation ratio, stratification variables, and randomly permuted block sizes) and allows the sequence to be exported for implementation (33). The platform provides documentation on permuted-block and stratified randomization procedures, supporting transparent reporting and reproducible sequence generation. The randomization lists were generated by the researcher prior to participant assignment and without access to any identifying information (e.g., names or email addresses) stored on the Equiem platform.

#### 2.7.2 Concealment mechanism and implementation

The randomization sequence was generated by the principal researcher using Sealed Envelope prior to data collection, exported, and stored securely in Microsoft Excel. The list was shared with only two authorized Garden State staff members who had controlled access to the Equiem platform where participant details were stored

Allocation was implemented sequentially by a designated Garden State staff member who matched each participant's email address to the next available group assignment on the pre-generated randomization list. This was performed directly within the Equiem platform interface, where participants' names and emails were visible only during assignment. The staff followed the randomization list exactly, without alteration or discretion.

To maintain allocation concealment and independence of implementation, the principal researcher did not have access to participant identifiers (including email addresses) or the allocation list at any point during the assignment process. Upon completion of assignment (and prior to email dissemination), a second Garden State staff member independently verified the accuracy of allocations by cross-checking the final participant–group assignments against the original Sealed Envelope list (e.g., confirming each participant's assigned group corresponded to the correct sequence position).

#### 2.7.3 Binding

Blinding of participants and implementation staff was not feasible due to the nature of the intervention and the manual assignment process within the Equiem platform. Each participant received an email corresponding to their assigned incentive condition (financial, social, or control) and thus was aware of their own allocation. However, participants were not informed that alternative incentive options existed, which helped to minimize offline cross-group contamination. Emergency unblinding procedures were not applicable, as participants were aware of their assigned condition upon receiving the email invitation and there were no concealed allocations to unblind.

Awareness of other incentive conditions could have influenced participants' willingness to participate. For instance, participants in the control group might have been less inclined to engage if they knew others were receiving financial incentives. To reduce this potential source of bias, participants were asked at the beginning of the questionnaire to keep their study participation and incentive details confidential until contacted by the research team (see Supplementary File S1). This request was particularly relevant in shared work environments where colleagues might have received different email interventions.

Although allocation blinding was not possible, allocation concealment was maintained until the point of assignment, as the randomization sequence was inaccessible to both participants and platform staff prior to implementation. During data analysis, participant identifiers would be pseudonymized to ensure objectivity and minimize any potential bias in outcome assessment.

### 2.8 Interventions

#### 2.8.1 Nature of intervention

**Figure 2:**
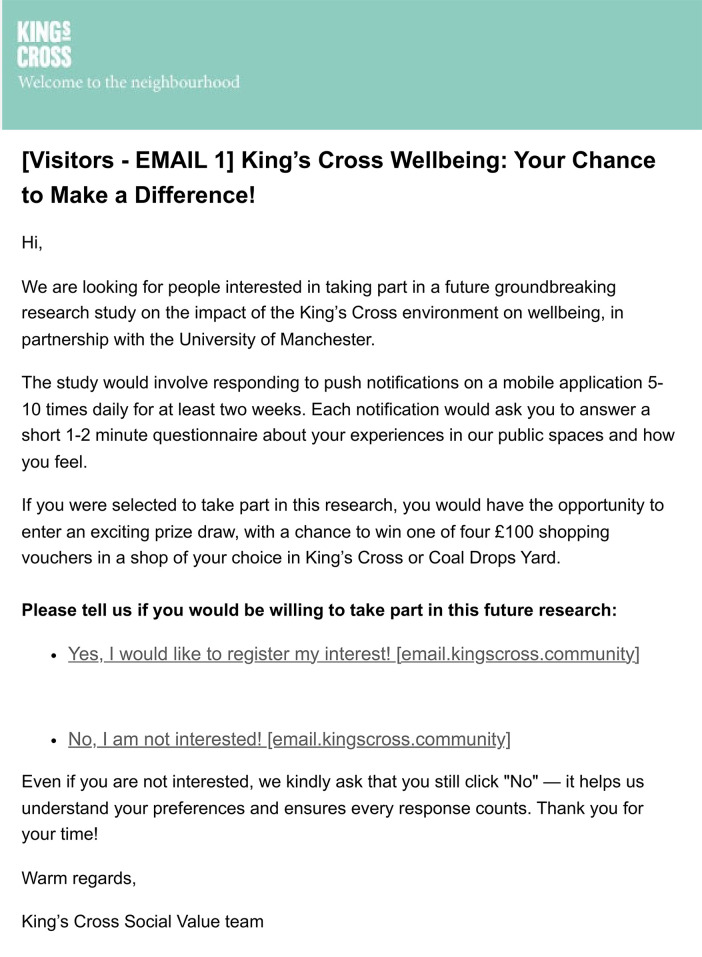
Example invitation email screenshot from the financial incentive condition. **Note.** The bracketed label in the example screenshot (e.g., “[Visitors - EMAIL 1]”) appeared only in internal test emails used to verify resident-type versions before distribution and was not included in participant-facing emails. The bracketed text shown after hyperlinks (e.g., “[email.kingscross.community]”) is an artifact of the internal screenshot examples and was not part of the intended participant-facing email wording. Exact internal screenshot examples from all trial arms across both email rounds, including the revised second-round reminder emails, are provided in Supplementary File 2.

This study tested whether the content of an email invitation—specifically, the inclusion of an incentive framing—affected recipients' willingness to register interest in a future smartphone-based EMA assessment of well-being in and around King's Cross. Eligible users were contacted via email through the King's Cross Equiem database as part of a neighborhood public-engagement initiative delivered in collaboration with a university. Each participant received one of three email versions: (1) financial incentive, (2) social incentive, or (3) no-incentive control. All emails used identical formatting and core wording; they differed only in a single paragraph describing the incentive (or no paragraph for the control condition). An example invitation email is shown in Figure 2. The condition-specific paragraph used in each trial arm in the first email round is summarized in Table 4, and exact screenshot examples from all trial arms across both email rounds are provided in Supplementary File S2. A second round of reminder emails was subsequently disseminated using revised wording and design while maintaining the arm-specific incentive framing. Full email materials are provided in Supplementary File S2.

**Table 4:** Condition-specific paragraph inserted into the invitation email for each trial arm in the first email round.

**Email component**	**Financial incentive condition**	**Social incentive condition**	**No incentive condition**
**Round 1: condition-specific paragraph**	If you were selected to take part in this research, you would have the opportunity to enter an exciting prize draw, with a chance to win one of four £100 shopping vouchers in a shop of your choice in King’s Cross or Coal Drops Yard.	If you were selected to take part in this research, you would have the opportunity to join the research team for an on-site social event in King’s Cross to discuss the research findings and meet with other participants.	No additional condition-specific paragraph.

The invitation described that the future EMA study would involve brief smartphone questionnaires about daily experiences in and around King's Cross, delivered multiple times per day over a two-week period. Recipients indicated willingness to participate via one of two embedded response options (Yes/No), each linking to a short follow-up questionnaire where responses were recorded securely.

In the financial incentive condition, the email stated that individuals selected for the future EMA study would be entered into a prize draw to win one of four £100 shopping vouchers redeemable at King's Cross or Coal Drops Yard. In the social incentive condition, the email stated that selected participants would be invited to an exclusive post-study event with other participants and researchers to discuss findings. The control condition included no incentive text.

To support participation, a single reminder email was sent to non-responders; the reminder maintained the same condition-specific incentive paragraph and call-to-action links, while using a brief reminder-oriented opening. The reminder email text is provided in Supplementary File S2.

#### 2.8.2 Delivery of intervention

The intervention emails were sent from the official email address of the King's Cross neighborhood's asset manager, The King's Cross Group, a familiar and trusted source for recipients due to their prior engagement with community communications (see Supplementary File S2). Successful email delivery was confirmed through the platform's delivery reports, and each participant received the intervention email twice: an initial email and a follow-up reminder.

The initial emails were disseminated on September 12, 2024, followed by a single reminder email sent one month later, on October 17, 2024; the participation survey link remained open until November 30, 2024. The need for a potential third reminder was evaluated; however, an additional round was not undertaken as it was deemed unnecessary. A one-time in-app smartphone notification to non-responders (linking to the same landing page) was also considered but not implemented to maintain email as the sole standardized delivery channel across conditions. Adding an in-app prompt could have changed contact salience/dose and introduced uneven exposure (e.g., disabled notifications or inactive app use), potentially confounding incentive effects, and could increase perceived burden. This decision, together with the decision not to send a third reminder, was informed by unsubscribe patterns observed after the initial email round, suggesting that additional contact beyond the single scheduled reminder could increase disengagement from the platform's routine communications (i.e., opting out/unsubscribing), rather than disengagement from the study itself.

At each dissemination round, participants who had already responded to the binary participation question were excluded from subsequent mailings to avoid redundancy. This process was conducted by two designated staff members at Garden State, who removed responders from the mailing list before each distribution. Yet, an apology wording was included in the reminder email as a courtesy fail-safe in the event that any prior responder received a reminder despite list cleaning. The reminder email differed only in the subject line and opening paragraph; all condition-specific content remained unchanged. All reminder emails were scheduled for delivery at 2:00 p.m. on the designated dates, based on prior communication analytics from The King's Cross Group, which indicated high open rates at that time

### 2.9 Study outcomes

#### 2.9.1 Primary outcome variable

The primary outcome variable of this study is the willingness to participate in a future smartphone ecological momentary assessment (EMA) study. The variable is measured through a binary response included in the intervention email. Participants could select either “*Yes, I would like to register my interest!”* (coded as 1) or “*No, I am not interested!”* (coded as 0). Operationally, “Yes” would be defined as completing the core demographic/residential relationship items and submitting a valid email address to register interest; “No” would be defined as selecting the non-interest option. Moreover, participants who did not respond to the invitation (i.e., did not click either option) would be coded as 0 (not willing) in the primary intention-to-treat analysis.

#### 2.9.2 Secondary outcome variable

The study also assessed two secondary outcomes among participants who indicated that they were unwilling to participate in the EMA study. The first secondary outcome captured the reason for unwillingness to participate. Participants were asked to identify one or more factors influencing their decisions not to participate. These factors included (1 = “Inadequate compensation”), app notification frequency (2 = “Too many notifications”), study duration (3 = “long/excessive study duration”), limited time availability (4 = “daily time constraints”), app download (5 = “Reluctance to install new app”), privacy concerns (6 = “privacy and data security concerns”), lack of interest (7 = “Lack of interest in the study”), and [8 = Other reasons(please specify)]. Participants were asked to “select the main reason(s) for not participating in the future research study” from this list.

The second secondary outcome assessed alternative incentive preference. This outcome was obtained only for participants who selected “*Inadequate compensation”* as their main reason for non-participation. These participants were subsequently presented with a list of seven potential incentive alternatives they preferred to be offered for a similar study in the future. They were asked, “Would any of these increase your likelihood to participate? Check all that apply” The incentive options were: nothing (1 = “Nothing will increase my likelihood”), guaranteed cash payment (2 = “£50 guaranteed cash on study completion”), gift card (3 = “Gift card on study completion”), prize draw (4 = “4× £100 cash prize draw”), personalized feedback (5 = “Personalized well-being feedback”), special event invitation (6 = “Exclusive post-research social & feedback event”), charity donation (7 = “Donation to charity”), and other (8 = “Other—please specify”).

### 2.10. Data collection

Data for this study were collected through online questionnaires hosted on the Give My View (GMV) platform, which was the pre-existing data collection system available for the project and therefore informed the implementation approach. GMV is a public citizen digital engagement platform developed by LandTech to facilitate community participation and feedback. Accordingly, the capabilities and limitations of the GMV platform, together with the randomized design of the study, informed the development of two-stage questionnaire combinations.

The email invitation contained two response links (“Yes”/“No”), each directing recipients to a corresponding questionnaire. Those selecting “Yes” completed a brief sociodemographic questionnaire capturing demographic characteristics and indicators of study interest; those selecting “No” completed a brief questionnaire capturing reasons for non-participation and incentive preferences (see Supplementary File S1).

To ensure responses were correctly attributed to the participant's assigned incentive condition and user category, separate questionnaire instances were created for each combination of incentive condition (financial, social, no-incentive), response option (Yes/No), and user category (residents, visitors, commercial occupiers), yielding 18 instances in total. Each instance was linked via a condition-specific URL and labeled with a structured identifier to support tracking and analysis (e.g., RFNY=Resident–Financial Incentive–Yes; VNICN=Visitor–No Incentive–No).

### 2.11. Data management

Several procedures were implemented to uphold data quality and ensure data security. The GMV survey design required responses for four core demographic items (gender, ethnic group, age range, and relationship to King's Cross), while follow-up preference items were optional. Additionally, the platform's device recognition feature guaranteed that once a participant submitted a response, they were unable to access the survey again, thereby preserving the uniqueness of each submission. As data were collected directly in a pre-coded digital format on the GMV platform, manual data entry was unnecessary, reducing the risk of transcription errors and enhancing the overall accuracy of the dataset.

The primary outcome—the participant's binary decision to participate (“Yes” or “No”)—was derived directly from the raw data exported from the GMV platform. Each survey response was coded post-collection: responses from participants who completed the “Yes” survey were assigned a value of 1 and those who completed the “No” survey were assigned a value of 0. This coding process ensured consistent and accurate representation of the primary outcome for analysis.

All data storage, handling, and sharing procedures will comply with institutional data security and ethical guidelines. Study data will be cleaned and anonymised prior to any sharing. Access to identifiable and cleaned study data will be restricted to authorised members of the research team and approved project partners in accordance with prior data-sharing agreements.

### 2.12. Analysis plan

The trial will be reported in accordance with the CONSORT guidelines for reporting randomized trials. The detailed statistical analysis plan has been published elsewhere (34). All analyses will follow the intention-to-treat principle, including all randomized invitees, to ensure a comprehensive assessment of the intervention's effectiveness (35). The primary analysis will compare the effectiveness of the three intervention arms. Secondary analyses will explore subgroup differences in response to the incentives as well as examine barriers to participation and alternative preferences. Sensitivity analyses will be conducted to evaluate the robustness of the findings. The following sections outline these analyses in detail.

#### 2.12.1. Primary outcome analysis

The primary outcome is willingness to participate (Yes/No), operationalized as submission of a completed core questionnaire and valid email address (“Yes”) vs. not registering interest (selecting the non-interest option or not selecting either option). The effect of incentive type (financial, social, or no incentive) on willingness to participate will be evaluated using logistic regression. The model will include incentive condition as the main predictor and residential status (residents, workers, visitors) as a covariate, consistent with the stratification used during randomization. Odds ratios (OR) with 98.33% confidence intervals (CI) will be reported to compare each incentive group with the control group. A two-tailed *p*-value of < 0.01667 will be considered statistically significant (Bonferroni-adjusted to control the family-wise error rate at 0.05 across the three planned pairwise comparisons). In addition to odds ratios, marginal risk differences (RD) will be estimated to enhance interpretability.

Predicted probabilities will be obtained from the fitted logistic model through marginal standardization and RDs will be computed as the absolute difference in these marginal probabilities between groups. Confident Intervals (95%) will be derived using robust standard errors or nonparametric bootstrap methods, as appropriate. Primary inferences for the three pairwise comparisons of incentive type on the primary outcome will use 98.33% confident intervals (CIs) to adjust for multiple comparisons. Exploratory subgroup analyses and complementary RD estimates use 95% CIs and will be interpreted as estimation rather than confirmatory hypothesis testing. Interaction terms in subgroup models will be evaluated at a two-tailed alpha level of 0.05 and results will be reported as exploratory findings. In addition to the frequentist primary analysis, Bayes factors will be calculated to complement interpretation of the primary outcome by quantifying evidence for differences between incentive conditions.

#### 2.12.2. Subgroup analyses

Exploratory subgroup analyses will assess whether the effectiveness of the incentive conditions varies across demographic and residential subgroups. Interaction terms between incentive type (financial, social, or none) and participant characteristics (residential status, gender, and age group) will be included in the logistic regression models. These analyses will evaluate whether certain incentives are more effective for particular subpopulations, such as specific residential groups or demographic categories, thereby identifying potential differences in responsiveness across participant types.

#### 2.12.3. Sensitivity analyses

Given the study design, some individuals who are not interested in the well-being study are expected not to click the “*No, I am not interested”* hyperlink to complete the corresponding survey, potentially resulting in missing data. To address potential bias from this nonresponse, sensitivity analyses will be performed to assess the robustness of findings (9).

These analyses will evaluate the potential impact of missing data on the primary outcome: willingness to participate in a future study. Because nonresponse is likely to be missing not at random (MNAR)—that is, individuals who are uninterested may simply close the email rather than actively opt out—alternative assumptions will be explored to assess how different missing-data mechanisms influence overall results (see Additional Document 2). By comparing results under these alternative assumptions with those from the main analysis, the sensitivity analyses will clarify the extent to which missing data may affect the estimated effectiveness of the incentives.

#### 2.12.4. Additional outcome analysis

##### 2.12.4.1. Analysis of barriers to participation

The first additional outcome analysis will focus on participants who decline to participate in the EMA study (i.e., those who select “No”). Each potential barrier to participation will be represented as a binary variable indicating whether a given reason was endorsed. Descriptive statistics will summarize the frequency and percentage of each reported reason, stratified by incentive condition. Graphical summaries (e.g., bar charts) will be used to visualize the most frequently cited reasons and their distribution across incentive conditions.

To examine whether endorsement of specific barriers varies by incentive type, separate Firth logistic regression models will be fitted for each reason, with incentive condition (financial, social, or no incentive) as the main predictor. Firth logistic regression will be used to reduce sparse-data bias and to address potential separation. Odds ratios (ORs) and 95% confidence intervals (CIs) will be reported for each incentive condition relative to the no-incentive group, and False Discovery Rate (FDR) adjustment will be applied across the set of reason-specific models to account for multiple testing.

The total number of reasons endorsed by each respondent will be analyzed as a count outcome using Poisson regression. A model including incentive condition as the main predictor will be fitted to assess whether the number of reported barriers differs across the financial, social, and no-incentive conditions. Incidence rate ratios (IRRs) and 95% confidence intervals (CIs) will be reported.

##### 2.12.4.2. Alternative incentives

The second additional outcome analysis will focus on participants who declined participation but indicated that specific alternative incentives might have increased their likelihood of taking part. Each alternative incentive option will be represented as a binary variable indicating whether that option was endorsed. Descriptive statistics will summarize endorsement frequencies, and graphical displays (e.g., bar charts) will be used to compare the distribution of endorsed options across incentive conditions.

Separate logistic regression models will be fitted for each alternative incentive option, with incentive condition (reference: no incentive) as the main predictor. Odds ratios (ORs) and 95% CIs will be reported, and FDR adjustment will be applied across the set of alternative-incentive models to control for multiple testing.

### 2.13. Monitoring

#### 2.13.1. Data monitoring

A formal Data Monitoring Committee (DMC) was not established because this is a low-risk, non-clinical study involving email invitations and voluntary online questionnaires, with no therapeutic or behavioral intervention and no anticipated harms requiring interim monitoring. Study conduct (including randomization and email dissemination) was overseen by designated staff at The King's Cross Group, and the research team monitored data completeness and quality throughout data collection. No interim analyses were planned.

#### 2.13.2. Harms

The study placed a strong emphasis on participant well-being and data security. Potential adverse effects were minimal, as the interventions are limited to digital communication and voluntary survey responses. Nonetheless, strict procedures were implemented to protect participants' privacy and maintain data confidentiality. All collected data were anonymized and stored securely in accordance with institutional and data protection policies. Participants were informed that their participation was voluntary and that they may withdraw at any time without consequence. Drop-out statistics were monitored through the *Give My View* platform, and a dedicated contact email was available to address participants' questions or concerns during the study. This ensured timely support and guidance in case any discomfort happened. The research team was committed to fostering a positive participant experience and took proactive steps to prevent stress or negative consequences related to study participation. Overall, the risk to participants' well-being was expected to be negligible.

#### 2.13.3. Auditing

Two staff at the asset manager's company will oversee the randomization and email dissemination. One member of staff will primarily be in charge, and a second member of staff will cross-check the protocol procedures to ensure they have been adequately fulfilled.

## 3 Discussion

Recruiting participants into ecological momentary assessment (EMA) studies remains a persistent barrier to scalability, especially when protocols require frequent prompts over sustained periods (7, 9). This protocol addresses a practical methodological gap by using a randomized controlled trial (RCT) to test two scalable recruitment strategies at the point of invitation: (i) a probabilistic financial incentive (prize draw for limited-value shopping vouchers) and (ii) a social incentive (an invitation to a community research feedback event). By experimentally comparing these approaches against a no-incentive control, the study will generate causal evidence on whether uncertain financial rewards or socially meaningful engagement opportunities are more effective in motivating enrollment into smartphone-based well-being research conducted in real-world community settings.

Another contribution is the study's focus on heterogeneity in recruitment effects. Stratified randomization by participant type, together with planned subgroup analyses by age and gender, will help identify whether incentive responsiveness differs across segments of the local population. This is important because recruitment strategies can unintentionally amplify participation biases if they preferentially attract particular groups. The study therefore has the potential to inform more inclusive recruitment frameworks—for example, if some groups are more responsive to collective recognition and social affiliation, whereas others prioritize immediate or tangible material benefit.

The study will also generate evidence on non-participation by systematically documenting reasons for declining. Understanding non-enrollment is critical because barriers such as perceived burden, privacy concerns, low perceived personal benefit, or lack of trust can be addressed through concrete design and communication changes. These data can guide more user-centered invitations and study procedures, improving transparency and reducing avoidable friction at the point of decision.

Relatedly, the study examines alternative incentive preferences among individuals who find the proposed incentives unappealing. Mapping preferences (e.g., guaranteed payments, personalized feedback, charitable donation, or other options) can inform the design of incentive models that better balance ethical, financial, and feasibility constraints, and can support iterative optimization of scalable recruitment strategies for future EMA studies.

Several potential limitations should be noted. Recruitment through an existing digital platform may introduce self-selection: individuals subscribed to community communications may differ from the broader population in digital access, socioeconomic characteristics, or baseline engagement with local initiatives. In addition, invitation wording can materially affect uptake; to minimize avoidable deterrence, future iterations should specify a clear maximum study duration and make incentive qualification criteria explicit. However, platform-based recruitment also offers a countervailing strength: access to a large, already-engaged population directly addresses a common bottleneck in EMA research—difficulty achieving adequate sample sizes under realistic field conditions. Finally, the primary outcome focuses on willingness to participate rather than sustained adherence, and willingness may not perfectly predict downstream engagement once EMA begins (36, 37). Nonetheless, willingness is a necessary precursor to participation and an ethically appropriate endpoint for evaluating recruitment strategies at the invitation stage.

Overall, this study will contribute scalable evidence on recruitment strategies for community-based smartphone EMA. By combining an RCT design with an embedded, real-world recruitment channel, it will provide important evidence on the causal impacts of incentive framing on participant uptake, thereby informing practical, equity-oriented approaches to recruitment in EMA research.

## 4 Ethics and dissemination

### 4.1 Research ethics approval

The study was approved by the University of Manchester Research Ethics Committee (Division: School Level Review) (Review reference: *2023-17042-30162*) on 30/06/2023. Given the nature of the project, the risk of participant distress was considered minimal, as participation required explicit opt-in consent and the study topic was not sensitive. Participants were reminded that they were free to stop the survey at any time and were not required to answer any questions they preferred to skip.

### 4.2 Consent

The Participant Information Sheet (PIS) was presented at the start of the GMV questionnaire as a series of paginated screens (see Supplementary File S1). After the PIS screens, participants were presented with an explicit consent checkbox (“I agree to take part in the study”), which was required before they could submit the questionnaire.

### 4.3 Confidentiality

All documents and data associated with the study, including reports, administrative files, and data collection outputs, were securely stored to maintain participant confidentiality. The study data were collected and stored on the *Give My View* survey platform under the researcher's official account. A copy of the dataset was downloaded and stored on the official university research computer, protected under institutional security protocols.

### 4.4 Access to data

The cleaned datasets will be accessible to the project's research team and the King's Cross neighborhood's development and asset management partner (The King's Cross Group), in accordance with prior agreements. The statistical analysis plan and related study materials are publicly available via the Open Science Framework (OSF) project page: https://doi.org/10.17605/OSF.IO/W3FVM An anonymized version of the dataset will also be archived and made publicly available via OSF. To ensure confidentiality, all shared datasets will be anonymized to remove identifiable participant information before access is granted or public release is made.

### 4.5 Dissemination policy

The study results will be disseminated to the target community to promote inclusiveness and inform future community research initiatives. Findings will also be submitted for publication in a peer-reviewed academic journal. Dissemination activities will prioritize transparency and accessibility, ensuring that both academic and non-academic audiences benefit from the study outcomes.
