# Supplementary material for: Examining the effectiveness of financial vs. social incentives to participate in a smartphone ecological momentary assessment well-being study: protocol for a randomized controlled trial
Source: Front Digit Health. 2026 Jun 12;8:1742191. doi: 10.3389/fdgth.2026.1742191 (PMC13303974; doi:10.3389/fdgth.2026.1742191)

After clicking any of the two options (“Yes, I want to Contribute” or “No, I am not able to participate at this time”) in the recruitment email, participants will be led to the following questionnaire hosted on GiveMyView platform.

**Questionnaire Text**

Hello! This short questionnaire is about the upcoming King's Cross study mentioned in the email. By completing this survey, you are expressing interest to take part in the study. Therefore, this is not an official invitation or consent to participate. If you are chosen to participate in the study, we will contact you again with the email you share with us here.

One last thing! We're researching various King's Cross groups. For accurate results, please keep the study confidential until we contact you further. Thank you in advance for your cooperation.

For more details, please click the following link: PARTICIPANT INFORMATION SHEET

I have read the Participant Information Sheet and consent to participate in this survey.

1. What is your gender?
   - - Male
     - Female
     - Other (please specify your other gender)
2. What is your ethnic group?
   - - White
     - Mixed ethnic background (e.g. White and Asian, White and Black African)
     - Asian or Asian British (includes Indian, Pakistani or Bangladeshi)
     - Black or Black British (includes Caribbean or African)
     - Chinese
     - Other ethnic group (please specify your other ethnic group)
3. Please select your age range
   1. 18 to 29
   2. 30 to 39
   3. 40 to 49
   4. 50 to 59
   5. 60 to 69
   6. 70 or above
4. What is your relationship with King’s Cross?
   1. I work in King’s Cross [skip to Question 5]
   2. I live in King’s Cross [skip to Question 6]
   3. I visit King’s Cross [skip to Question 7, 8, and 9]
5. Which building category do you work in?
   1. Office occupier
   2. Retailer occupier
   3. Other (please specify your work employer, i.e., name of employer)
6. Do you or your household own or rent your accommodation within King’s Cross?
   1. I own my home outright
   2. I am buying with mortgage/loan
   3. I am renting
   4. I live rent-free
   5. Lives in the house rent-free
7. How often do you typically visit King's Cross?
   1. Daily
   2. Weekly
   3. Monthly
   4. Few times a year
8. What specific activities bring you to King's Cross? (Select all that apply)
   1. Work
   2. Socialising
   3. Shopping
   4. Dining/Restaurant
   5. Entertainment (e.g., movie concert)
   6. Other (please specify)
9. On average, how much time do you spend during each visit to King's Cross?
   1. Less than one hour
   2. 1-2 hours
   3. 2-4 hours
   4. More than 4 hours
10. What's your best email? We'll contact you when ready to launch the future study.

After completing the above questionnaire and clicking “submit”, participants will be asked the follow-up questions presented next.

**Clarification on required vs optional items:** Questions 1–4 (core demographics and relationship to King’s Cross) were set as required to minimize missingness in key covariates. Questions 7–10 (visitor/workplace follow-ups and contact email) were presented based on the skip logic shown above; items 7–9 were optional and could be skipped, while Question 10 (email address for future contact) was required for participants who selected “Yes, I would like to register my interest.” The subsequent preference questions shown below (asked only to participants who selected “Yes”) were optional.

Questions for only participants who answer “Yes, I want to contribute”.

*Thank you for your responses. Only 3 questions remain. To assist in planning the future study in King's Cross, please specify the options you would be willing to participate in as part of the study.*

*If you are chosen to participate in the study, how would you prefer to get the research app on your phone to answer the study's quick surveys?*

*Please specify your preferred method to get the research app on your phone.*

- 1. Download from app store
  2. Download a website as an app (quicker download, lesser storage, offline functionality, app-like experience)
  3. I don’t mind either option

*If you are chosen to participate in the study, how would you like to share your phone’s location while completing the daily survey?*

- 1. Select nearest location photo
  2. Tap “yes” for each GPS prompt
  3. Allow auto-track in background
  4. I have no specific preference
  5. Not willing to share location
  6. Manually type your location

*If you are chosen to participate in the study, how many times would you like to **receive notifications on your phone daily** to complete quick surveys?*

1. 2 times a day
2. 4 times a day
3. 6 times a day
4. I don’t mind

Questions for only participants who answer “No, I am unable to participate at this time”.

*Thank you for your feedback.*

*To help us understand how we could better engage our community in research, please indicate whether any of the following options would change your participating decision.*

Please select your 1-4 main reasons for not participating in the future research

- 1. **Inadequate compensation**
  2. **Many notifications (Too many notifications)**
  3. **Excessive study duration (Long/excessive study duration)**
  4. **Daily time constraints**
  5. **Don’t want to install new app (Reluctance to install new app.)**
  6. **Privacy/data security concerns**
  7. **Lack of interest (Lack of interest in the study)**
  8. **Other reason (please specify the other reason(s))**

Would any of these increase your likelihood to participate? Check all that apply

1. **Nothing will increase my likelihood.**
2. **£50 guaranteed cash on study completion**
3. **Gift card on study completion**
4. **1 of 4 £100 cash prize draw**
5. **Personal wellbeing feedback**
6. **Special event invitation: exclusive post-research social & feedback event**
7. **Donation to Charity**
8. **Other (please specify)**

**Screenshot of Questionnaire on the Give My View platform**

Questionnaire for unwilling participants


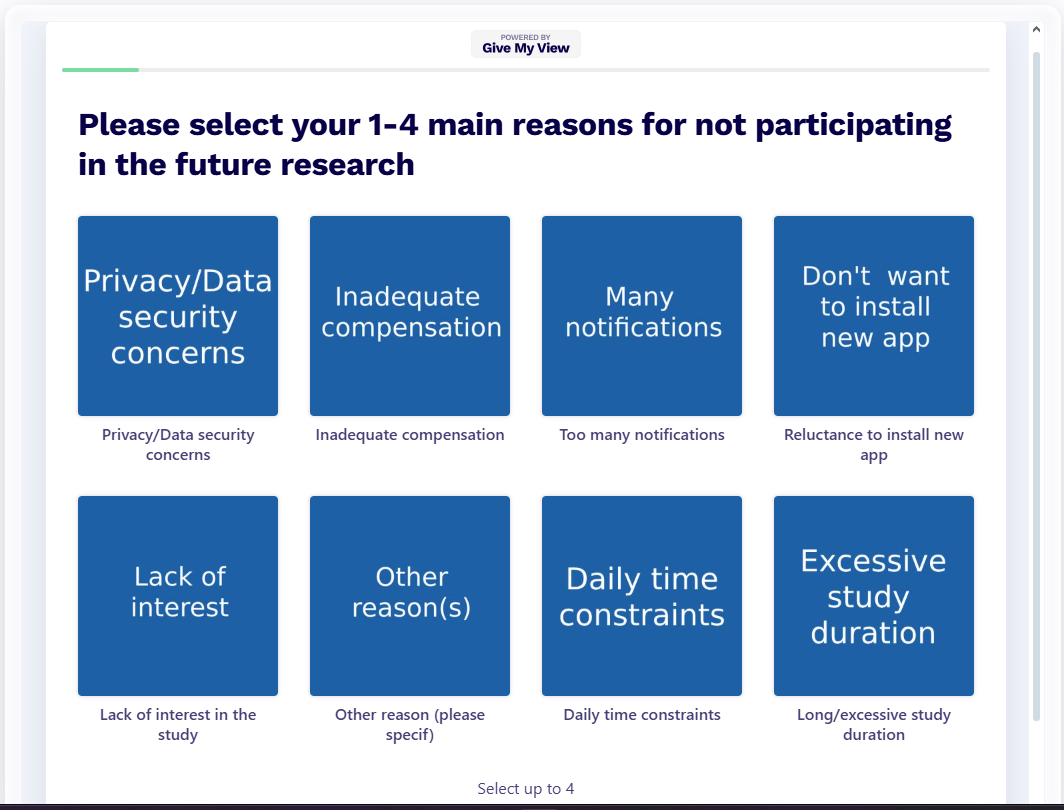


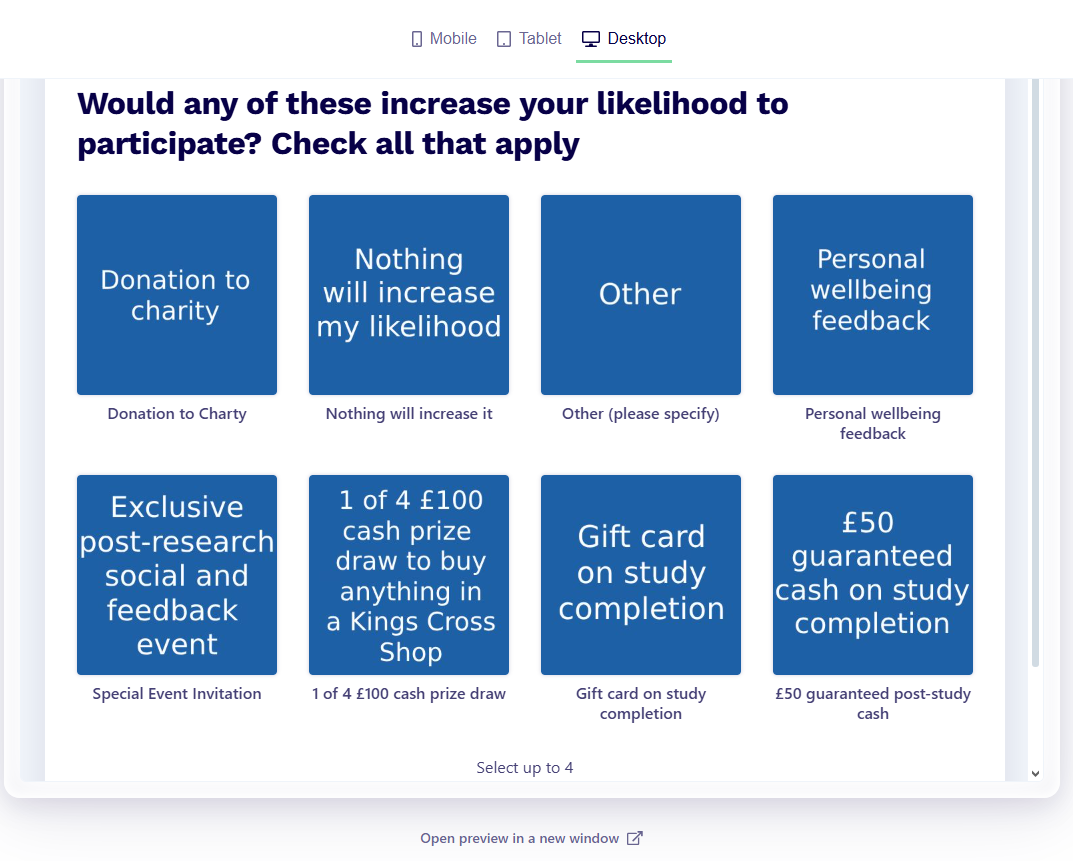


**Questionnaire for willing participants**


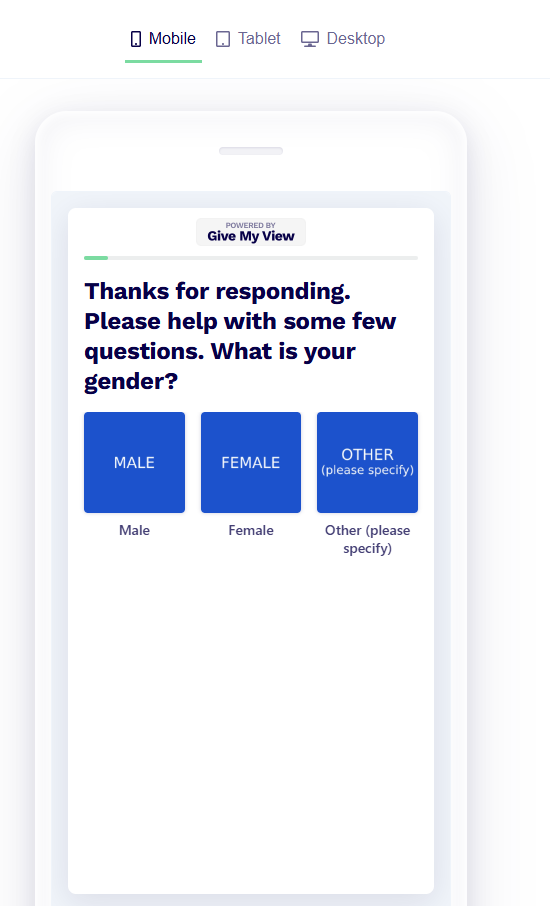


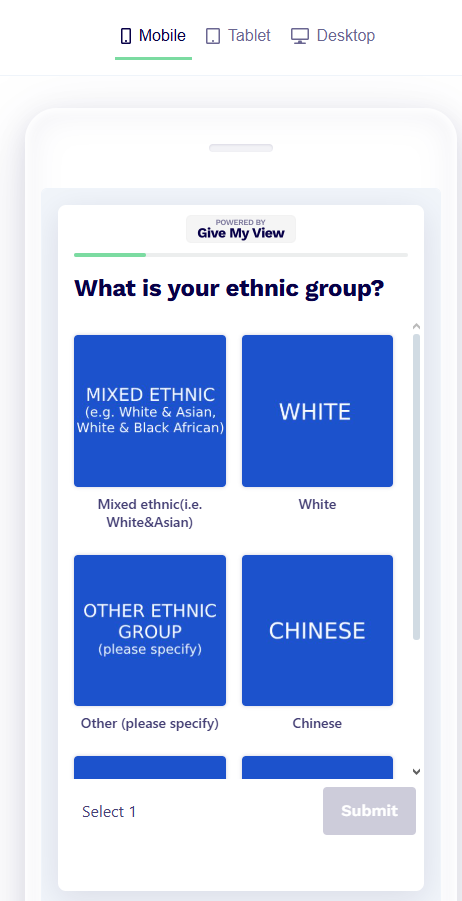


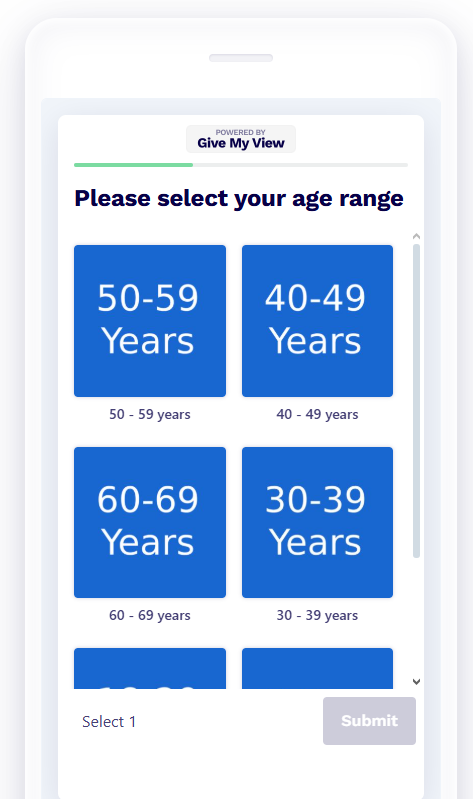


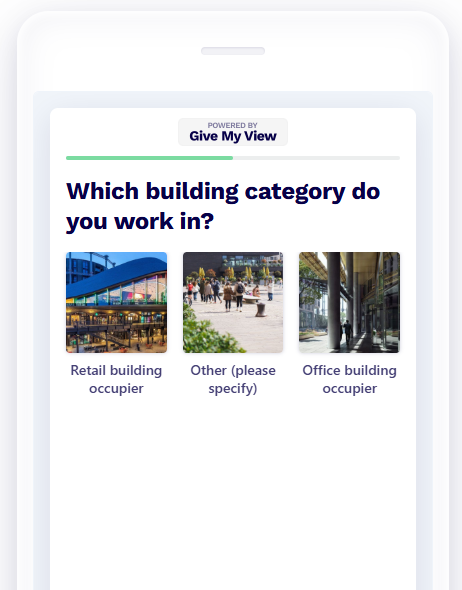


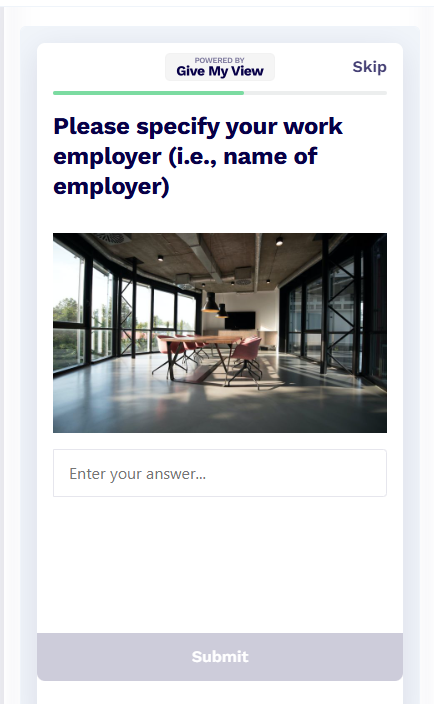


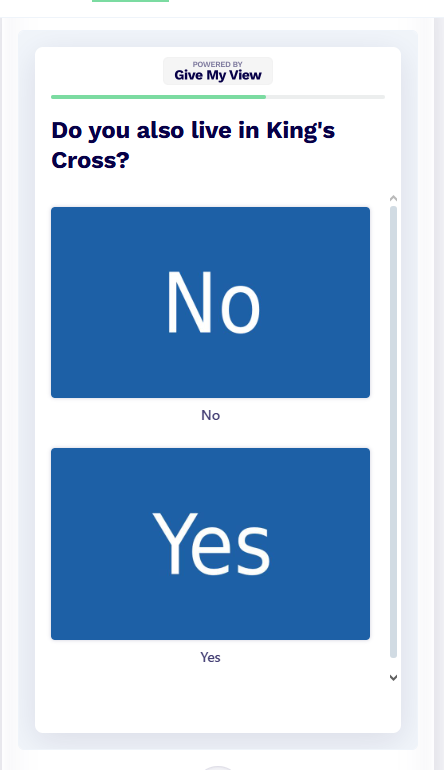


If yes , then:


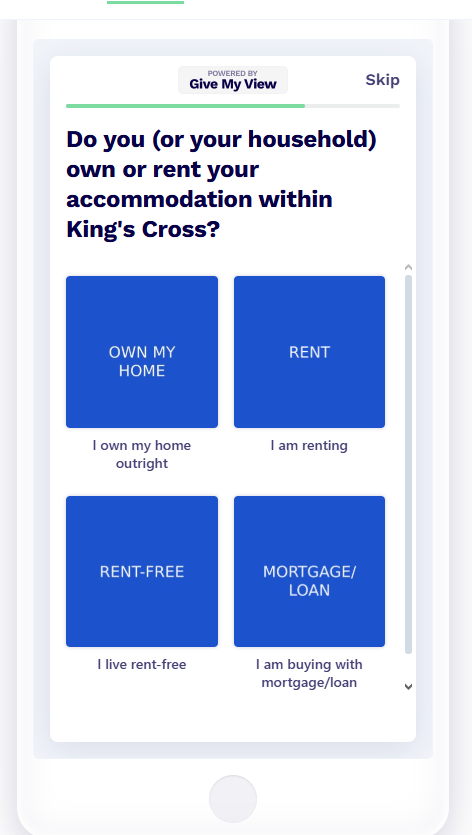


If no, then:


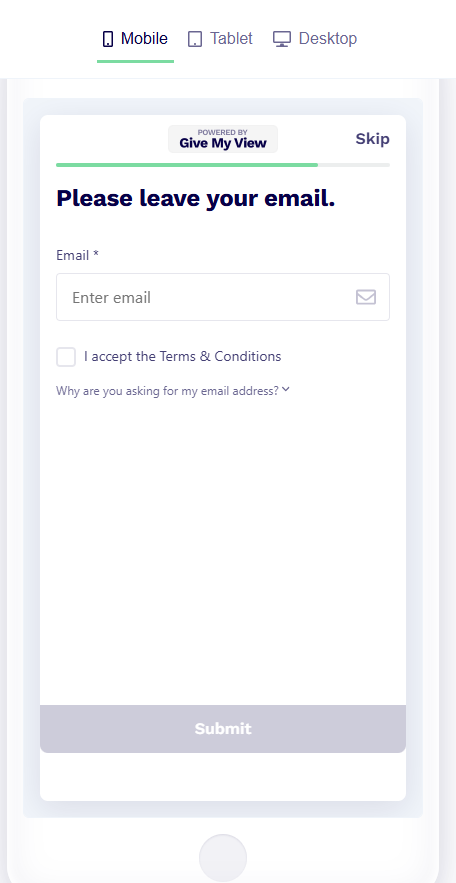


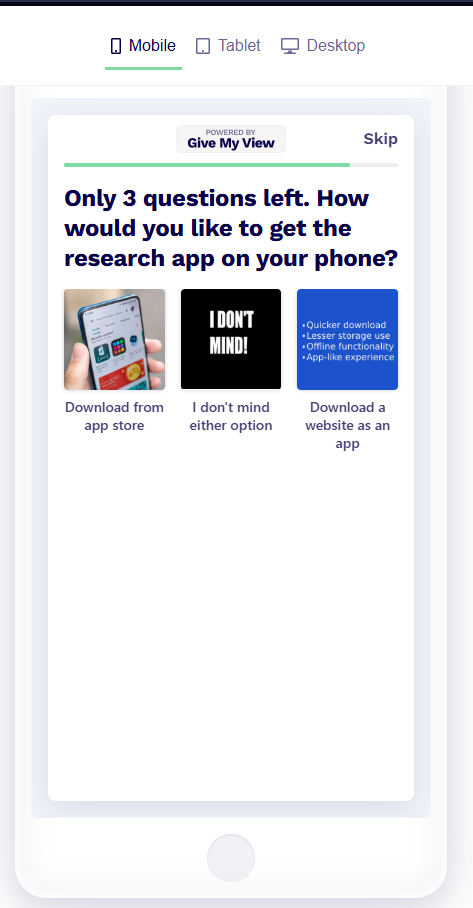


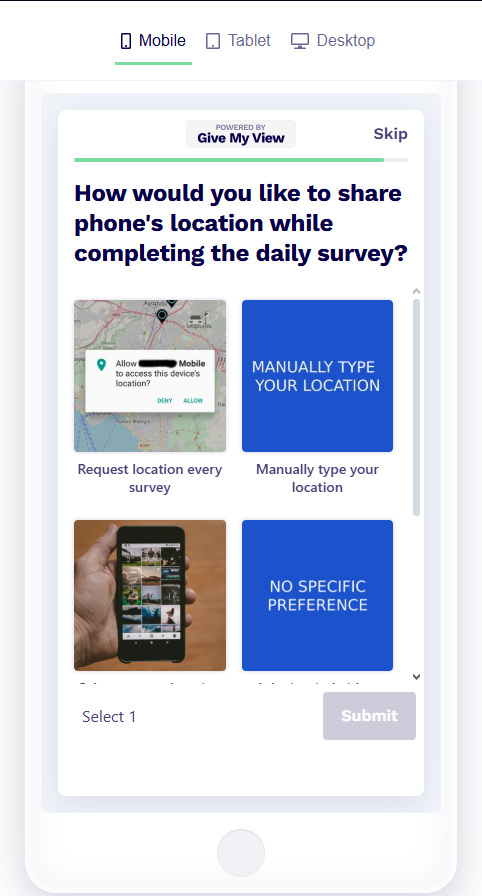


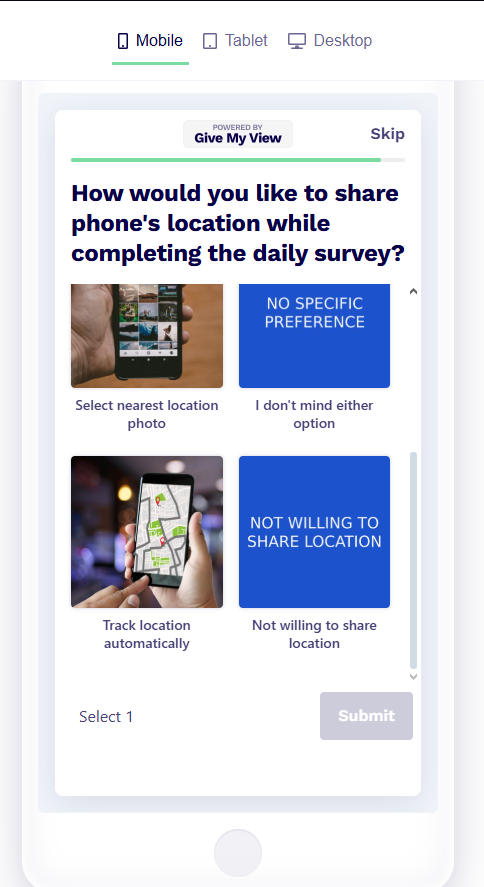


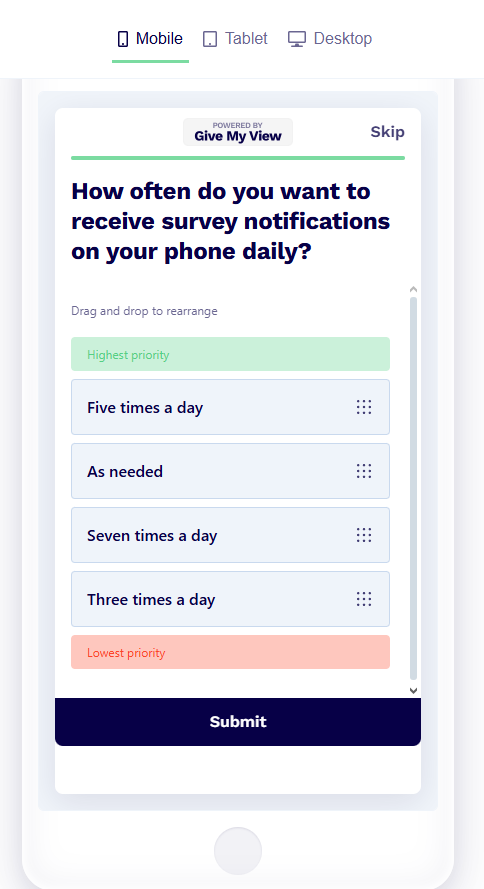


VISITORS


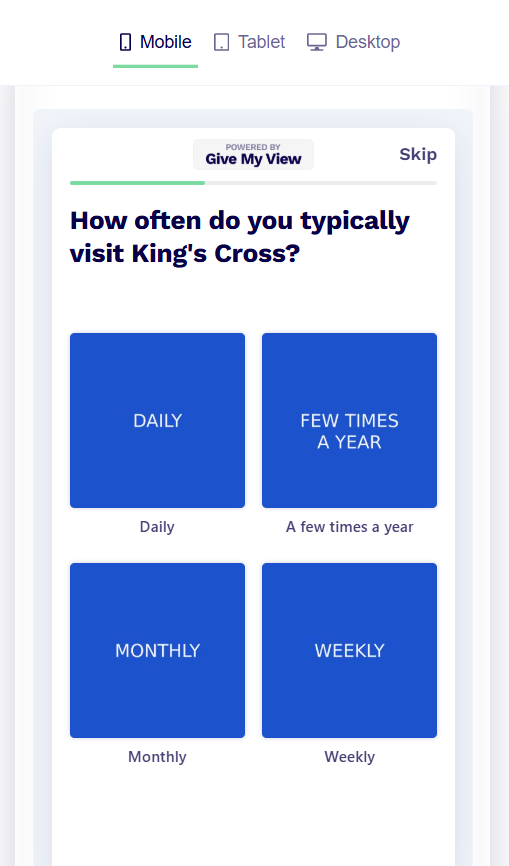


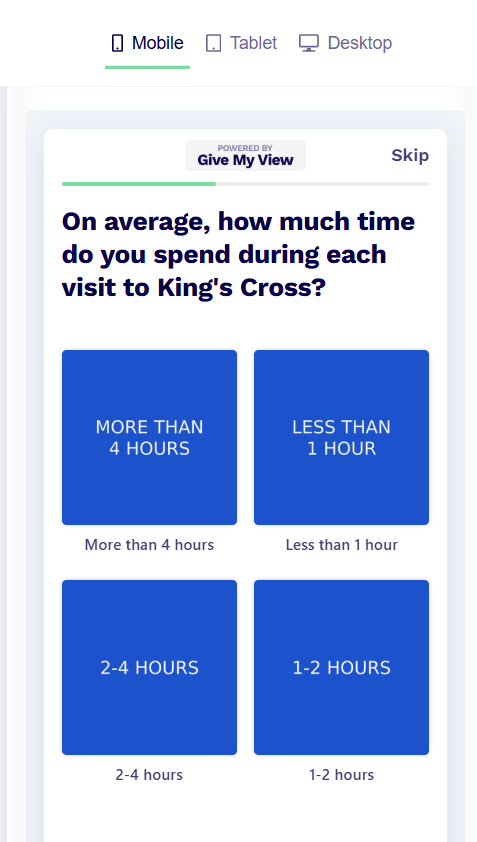


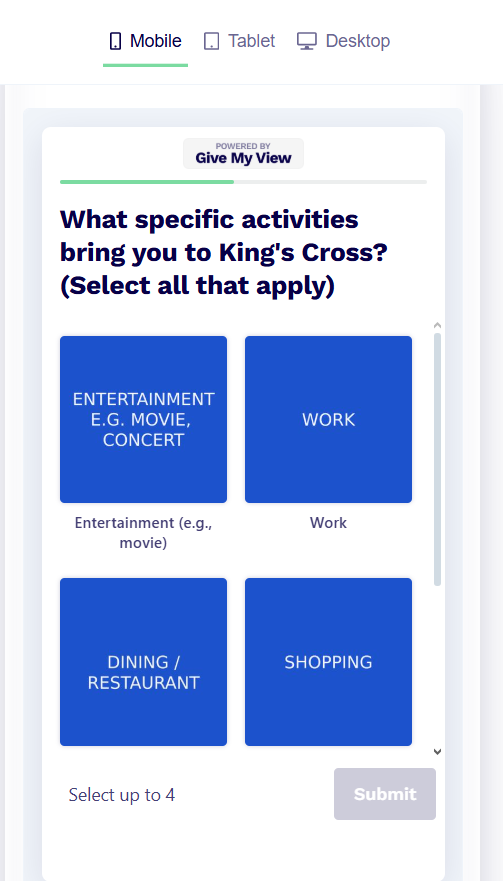

Supplement: Supplementary file 1 [file supplementaryfile1.docx]
