# Supplementary material for: Examining the effectiveness of financial vs. social incentives to participate in a smartphone ecological momentary assessment well-being study: protocol for a randomized controlled trial
Source: Front Digit Health. 2026 Jun 12;8:1742191. doi: 10.3389/fdgth.2026.1742191 (PMC13303974; doi:10.3389/fdgth.2026.1742191)
Supplement: Supplementary file 2 [file supplementaryfile2.docx]

**Screenshots of emails sent to respondents**

**Prize Draw 1^st^ round**


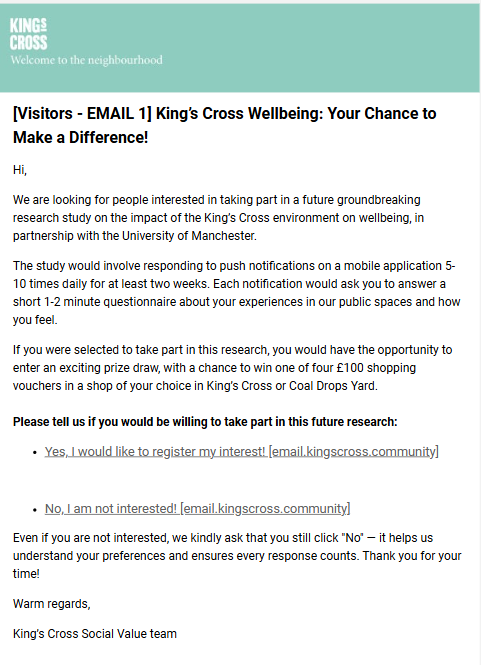


**Social Incentive 1^st^ round**


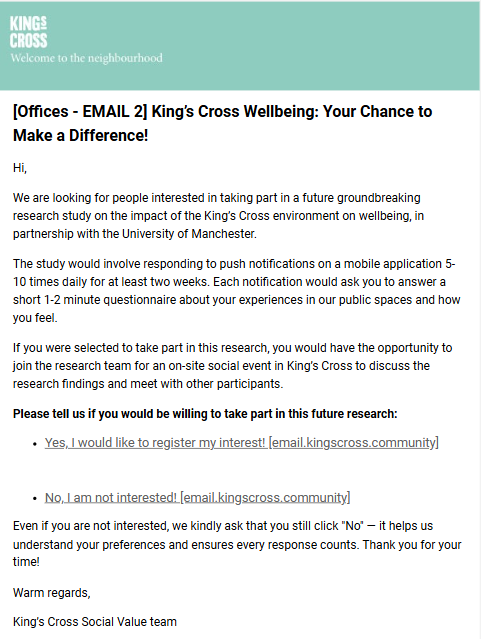


**Prize Draw 2^nd^ round (Follow-up design)**

Note: Although prior responders were removed from reminder mailings, the reminder template retained a brief apology as a fail-safe in the event of inadvertent re-contact.


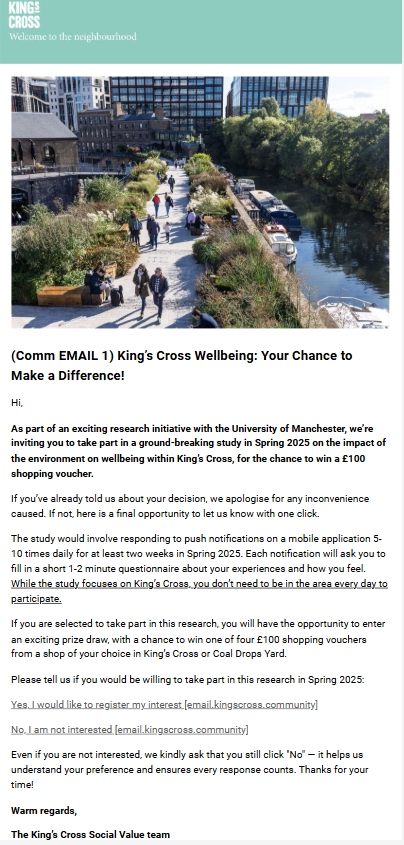


To view screenshots of the exhaustive email combinations sent out to each resident group in high resolution, please visit the Open Science Framework (OSF) project’s repository: <https://doi.org/10.17605/OSF.IO/W3FVM>
